# Supplementary material for: Reduced Staphylococcus Abundance Characterizes the Lesional Microbiome of Actinic Keratosis Patients after Field-Directed Therapies
Source: Microbiol Spectr. 2023 May 22;11(3):e04401-22. doi: 10.1128/spectrum.04401-22 (PMC10269920; doi:10.1128/spectrum.04401-22)
Supplement: Supplemental file 1 — Figures S1-S3. Download spectrum.04401-22-s0001.pdf, PDF file, 0.8 MB [file spectrum.04401-22-s0001.pdf]

Supplemental Fig. S1

week 24

LEfSe analysis CAP vs Diclophenac LDA > 2

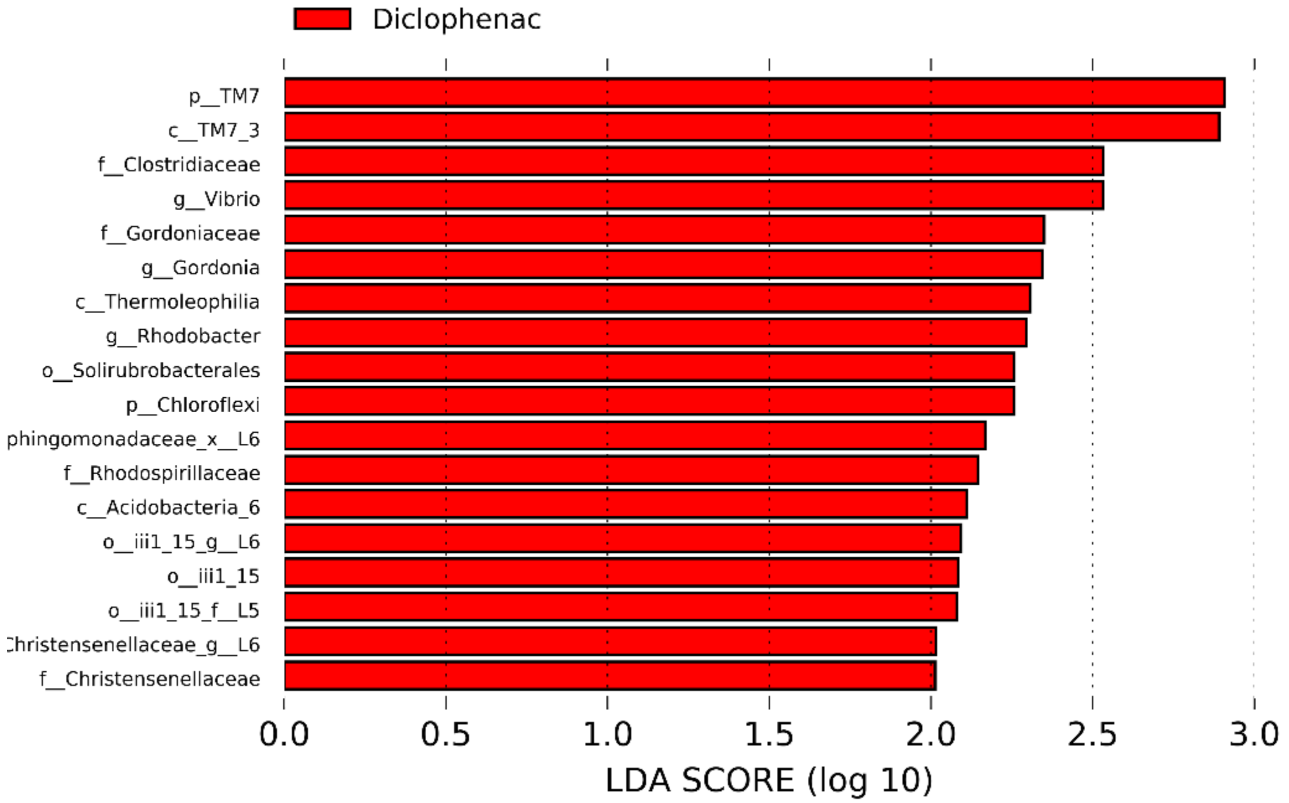

week 36

LEfSe analysis CAP vs Diclophenac LDA > 2

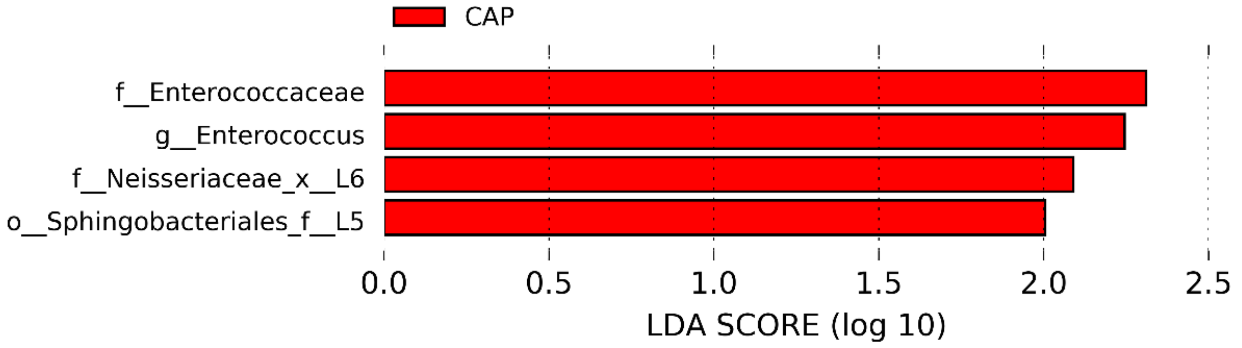

Linear discriminant effect size (LEfSe) analysis for identification of bacterial taxa enriched in diclofenac (DIC) or cold atmospheric plasma (CAP) treated patients. A Differential taxa in capillitium samples at week 24 with LDA score > 2 were enriched in DIC treated patients, no taxon was enriched in CAP treated patients at week 24. B Differential taxa in capillitium samples at week 36 with LDA score > 2 were enriched in CAP treated patients, no taxon was enriched in DIC treated patients at week 36.

Supplemental Fig. S2 **Timepoint Treatment**

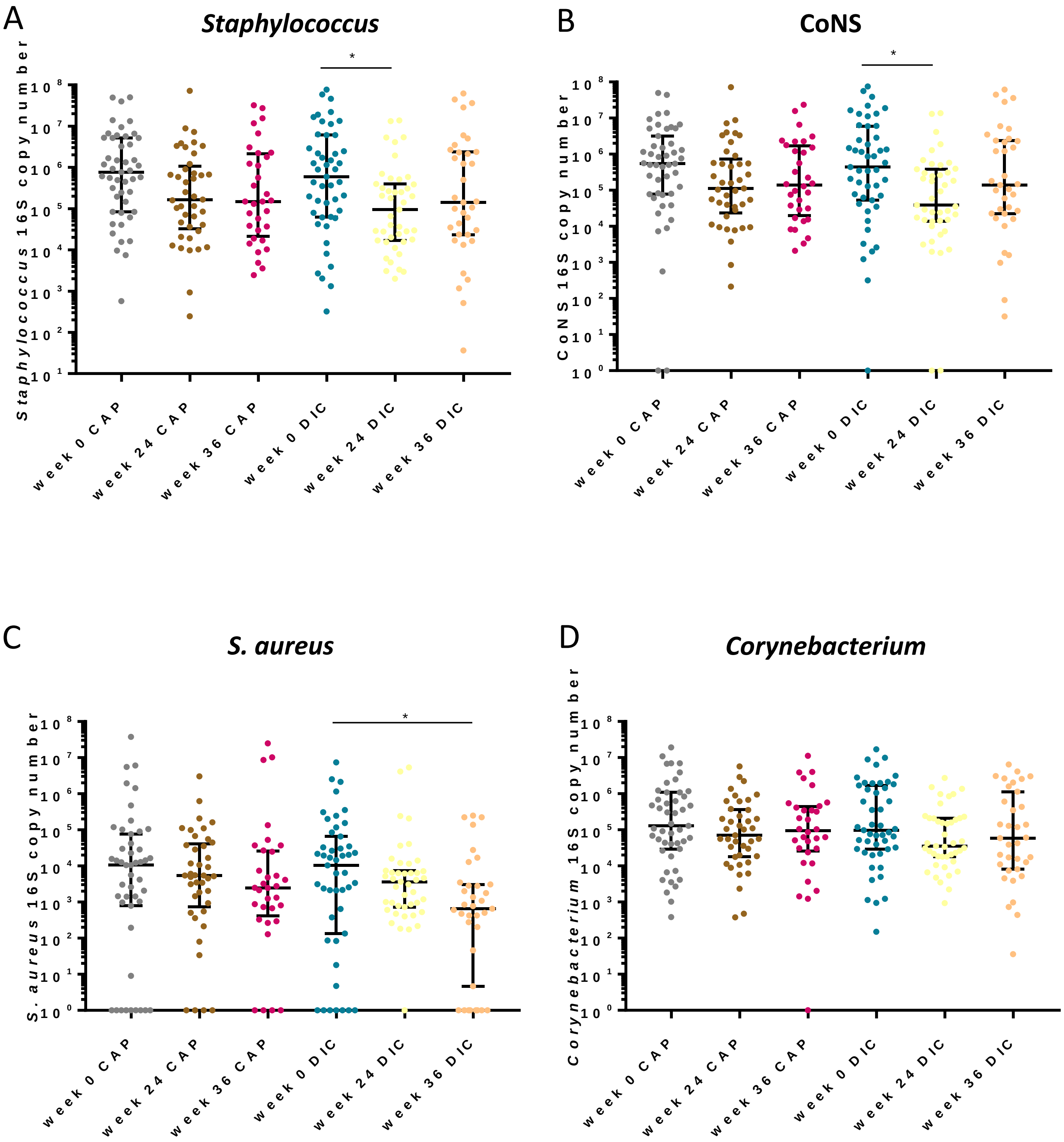

Absolute *16S rRNA* gene copy number from capillitium skin swab samples linked to timepoint and treatment of **A** *Staphylococcus* genus, **B** coagulase negative staphylococci (CoNS), **C** *S. aureus*, **D** *Corynebacterium*. Bars indicate median ± interquartile range.

Supplemental Fig. S3

Treatment response week 24

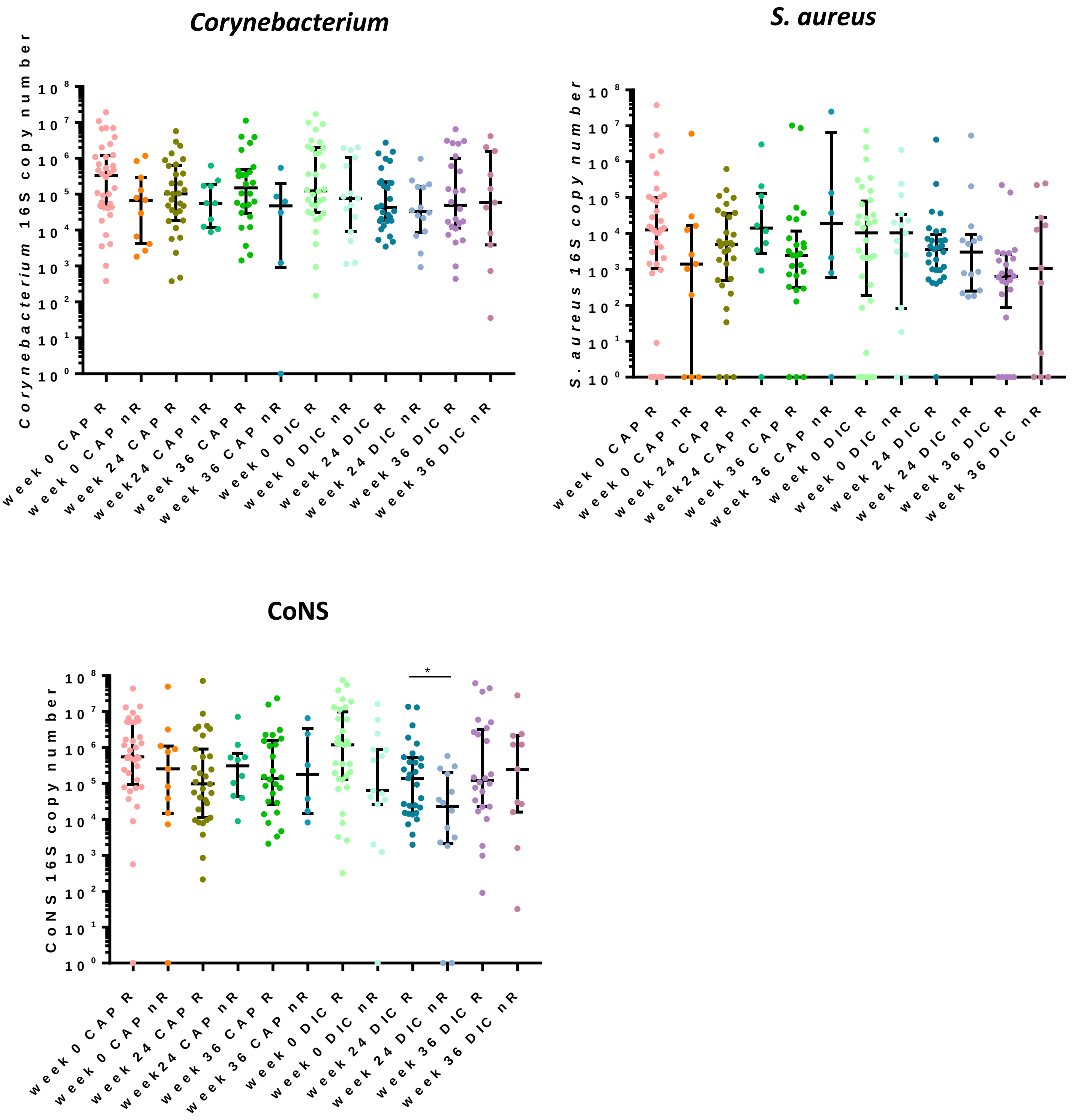

Absolute *16S rRNA* gene copy number from capillitium skin swab samples linked to treatment response to the respective treatment at week 24 for R versus nR at that time point. The relative abundance is shown for week 0, 24 and 36 in CAP and DIC treated patients of **A** *S. aureus*, **B** coagulase negative staphylococci (CoNS), **C** *Corynebacterium* genus. Bars indicate median ± interquartile range. Mann-Whitney U test was performed to test for significant differences between R and nR at week 24 for the respective timepoint and treatment.
